# Evaluation of Automatic Blood Analyzer as Screening Method in Fetomaternal Hemorrhage

**DOI:** 10.1155/2019/6481654

**Published:** 2019-02-26

**Authors:** Marcella R. Cardoso, Caroline N. de Souza-Araújo, Maria Cecília R. Talarico, Juliana Heinrich-Mouçouçah, Fernando Guimarães, Ricardo Barini

**Affiliations:** ^1^Department of Obstetrics and Gynecology, University of Campinas School of Medicine, Rua Alexander Fleming 101, 13083-891 Campinas, Brazil; ^2^Women's Hospital Prof. Dr. José Aristodemo Pinotti, Centro de Atenção Integral à Saúde da Mulher (CAISM), University of Campinas, Rua Alexander Fleming 101, 13083-891 Campinas, Brazil

## Abstract

Screening of fetomaternal hemorrhage (FMH) is essential in management of fetomaternal antigen incompatibilities of blood. The objective in this study was to evaluate the ability of automatic blood analyzer (ABA) to screen FMH, also comparing this method with flow cytometry (FCM). The contents of fetal red blood cells and fetal hemoglobin were evaluated by FCM and ABA, respectively, using both blood samples of male adults laced with umbilical cord blood diluted at 1/10, 1/100, 1/1,000, and 1/10,000, or blood from puerperal women collected within 48 hours following delivery. FCM had better performance (area under curve, AUC = 0.8723) than ABA (AUC = 0.6569) in detecting fetal blood laced with blood from male adults. At a critical level of 0.5%, ABA indicated that 27.5% of puerperal women would have FMH while FCM did not detect FMH. Our results showed that ABA overestimates FMH and disagrees with FCM on indicating puerperal women with FMH. ABA is inadequate for being used to screen for or to measure FMH.

## 1. Introduction

A correct and rapid detection and quantification of fetomaternal hemorrhage (FMH) is essential for the management and treatment of RhD discordant pregnancies. The amount of fetal blood transferred into the maternal blood circulation determines the prophylactic dose of anti-D immunoglobulin to prevent isoimmunization during pregnancy and postpartum [1].

FMH is the process of transferring blood from the fetus to the maternal intravascular compartment due to chorionic villus bleeding. Isoimmunization may occur in a RhD negative mother carrying a RhD positive fetus. This may lead to the production of anti-D antibody causing the development of hemolytic disease and severe anemia of the fetus and newborns [2–4]. The purpose of prophylaxis is to prevent RhD isoimmunization in future pregnancies. However, prophylaxis is based on the administration of standard doses of anti-D immunoglobulin which frequently corresponds to an overtreatment of the patient. Adequate doses of immunoglobulin rely on the accurate evaluation of the amounts of fetal blood in women's circulation, using methods with limited availability [5]. 

The first method developed for FMH quantification is a technique based on acid elution of blood smear, known as Kleihauer-Betke test [6], which is currently obsolete [6–9]. Flow cytometry (FCM) has become the standard method for detecting and measuring FMH and is commercially available as an assay kit. FCM combines cell morphology and conjugates of fluorophore molecules with monoclonal antibodies for multiparametric analysis of specific cell population. FCM evaluation of FMH is achieved by using anti-HbF and anti-RhD antibodies to identify a specific group of blood antigens that act as markers for red blood cells (RBC) from fetus. Anti-HbF monoclonal antibodies allow the discrimination of three distinct cell populations: fetal RBCs, F-cells, and adult RBCs. FCM identification of F-cells eliminates the disadvantage of Kleihauer-Betke [10–12].

Automatic blood analyzers (ABA) combine different analytical procedures into a single device, allowing rapid determination of multiple blood parameters using a small sample volume. Automatic blood analyzer is used to define standards of accuracy, reliability, and performance of blood gas tests by measuring parameters of pH, electrolytes, metabolites, and oximetry using spectrophotometry technology as in the case of fetal hemoglobin dosage [13]. Since it discriminates adult and fetal hemoglobin, this device is potentially useful for the evaluation of FMH. Additionally, ABA would represent a quicker and lower-cost alternative for the assessment of FMH compared to Kleihauer-Betke and/or FCM [14]. The objective in this study was to evaluate the ability of ABA to screen for FMH at critical level of 0.5% which is within the hemorrhage volumes neutralized by standard doses of anti-D treatment, also comparing ABA with FCM, a method that has been replacing Kleihauer-Betke test for this end.

## 2. Subject and Methods

### 2.1. Subjects and Blood Collection

Blood samples were collected from 57 male adults weighing more than 50 kg, who denied hematological pathologies and metabolic diseases. Blood samples from 14 newborns weighing above 2.5kg were collected from the umbilical cord just after birth. Additionally, blood samples from 40 puerperal women were collected within 48 hours following delivery at Hospital da Mulher Prof. Dr. José Aristodemo Pinotti (CAISM), Women's Hospital at University of Campinas (UNICAMP, Campinas, Brazil). All blood samples of umbilical cord or puerperal women were collected from subjects who denied metabolic or hematological diseases and delivered without obstetric complications. Blood collections were performed using 10ml vacuum blood-sampling tubes containing sodium heparin (Vacuette, Campinas, Brazil). The study was approved by the Ethical Review Board, Research Ethics Committee of UNICAMP (1.386.253/2016), and was registered on the Brazilian National Health Council (CAAE: 49989315.0.000.5404). Subjects or their legal guardian received information about the study and gave written informed consent before blood sampling.

### 2.2. Blood Sample Preparation

Blood samples containing fetal blood were prepared by serially diluting blood taken from umbilical cord with blood of male adults. Initially, 50*µ*l of umbilical cord blood was laced in 450*µ*l of adult blood. Subsequently, a tenfold dilution factor was used, always transferring 50ul for each dilution step and resulting in blood samples containing fetal blood at dilutions of 1/10, 1/100, 1/1,000, and 1/10,000. This procedure resulted in 285 artificially diluted blood samples, containing from 1 to 1,000 fetal red blood cells per 10,000 adult red blood cells. In addition, undiluted blood samples of adults and umbilical cord were also evaluated as negative and positive controls, respectively. The samples were thoroughly homogenized during the dilution process and prior to analysis. Blood samples from puerperal women were used without any previous preparation.

### 2.3. Blood Sample Evaluations

The contents of fetal red blood cells and fetal hemoglobin in all blood samples (blood samples from male adults, umbilical cord, puerperal women, and serially diluted fetal blood samples) were evaluated by FCM and ABA, respectively. The FMH QuikQuant kit (IQ Products, Groningen, ME) was employed for the preparation of blood samples prior FCM based analysis, following the manufacturer's recommendations. Initially, serially diluted blood samples prepared as aforementioned and blood samples from puerperal women were diluted at 1/20 ratio with QuikQuant buffer. Then, 10*μ*l of diluted blood samples was fixed by addition of 0.75ml of glutaraldehyde (0.04% in PBS; Sigma-Aldrich, Saint Louis, MO). During the period of fixation (10 minutes at room temperature), samples were intermittently vortexed. Thereafter, 1.5ml of Trillium IntraCell ™ working solution was added to each samples tube, which remained incubated at room temperature for 10 minutes. Samples were washed by centrifugation and the supernatants were removed. Cell pellets were suspended with 10*μ*l of Trillium FMH QuikQuant ™ reagent, which contains fluorescein isothiocyanate-conjugated anti-HbF and propidium iodide, and 40*μ*l QuikQuant ™ FMH Buffer working solution to prior incubation for 10 minutes in the dark. After the incubation period, samples were washed twice by centrifugation. Data acquisition was performed using a FACSVerse cytometer with FACSuite software (Becton Dickinson, San Jose, CA, USA) and a minimum of 50,000 cells were acquired. Data analysis was conducted using FlowJo software (Version 10; Tree Star, Ashland, OR, USA).

In parallel, the same blood samples were analyzed on an ABA ABL800 flex (Radiometer Medical, Brønshøj, Denmark). In this equipment, fetal hemoglobin is assessed by technics based on oximetry and spectrophotometry principles and does not require previous preparation of blood sample.

### 2.4. Statistics

The profile of the samples according to the variables under study was assessed by descriptive statistics. The accuracy of FCM and ABA to detect fetal blood laced with blood samples from male adults was further evaluated by plotting the receiver operating characteristic (ROC) curves for each of the methods. In order to analyze the relationship between the amounts of fetal blood detected by the two methods (FCM and ABA) the Spearman correlation coefficient was calculated. Because results included values equal to zero, all the values obtained by ABA and FCM were transformed as log_10_ (x + 0.5). Logarithmic values were also used for analyzing the agreement between the FCM and ABA by Bland-Altman method. The level of significance was set at p value < 0.05. Statistical calculations were performed using Prism 6 (GaphPad Software; La Jolla, CA, USA) and the Statistical Analysis System (SAS) for Windows, version 9.2 (SAS Institute Inc., 2002-2008, Cary, NC, USA).

## 3. Results

### 3.1. Evaluation of Fetal Blood in Serially Diluted Blood Samples

**Figure 1:**
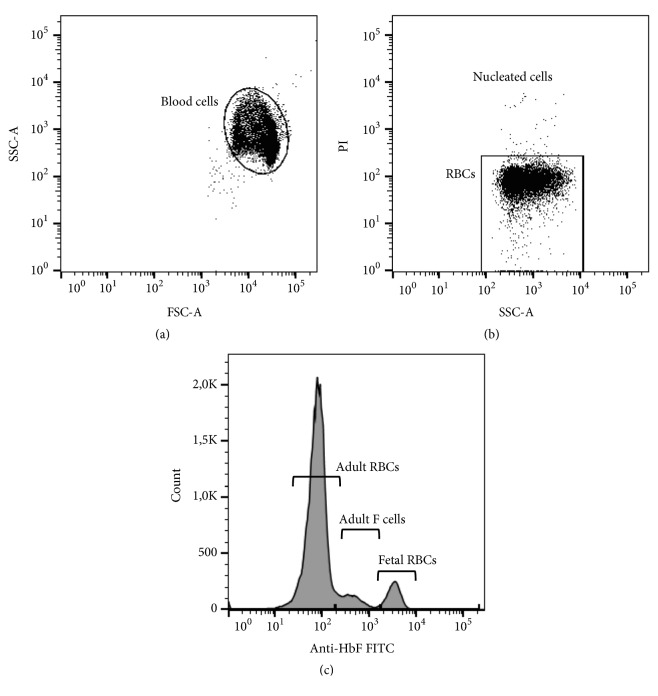
Flow cytometry-based analysis of blood samples, showing the gating strategy. The figure shows a representative sample at 1/10 dilution of umbilical cord blood in blood of male adult. (a) Total blood cells were gated on logarithmic dot plot; (b) nucleated cells were excluded by gating the propidium iodide (PI) negative cell population. (c) Different red blood cell (RBC) populations were detected and quantified based on their fetal hemoglobin content. In addition to histogram, events within the gate of fetal RBCs were also evaluated by plotting the fluorescein isothiocyanate (FITC) channel against phycoerythrin (PE) and side-scatter (SSC) channels to confirm the percentage of events.

The main characteristics of the subjects at the time of blood sampling were shown in Table 1, while Figure 1 depicts the gating strategy used in flow cytometry-based analysis to quantify fetal red blood cells in blood samples.

Both methods were able to differentiate between samples with and without fetal blood, while assessing blood samples of male adults containing fetal blood. However, this capacity was limited as dilution of fetal blood increased. Thus, ABA results were significantly different between samples with and without fetal blood up to dilution 1/10, while FCM results were significantly different up to dilution 1/100 (Supplementary Table 1).

**Figure 2:**
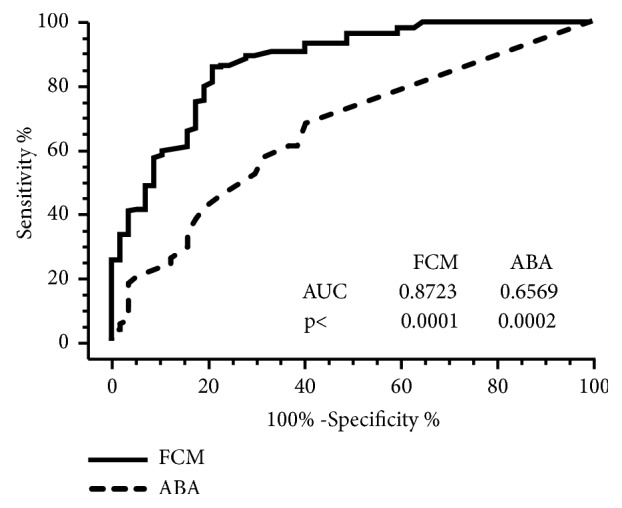
Receiver operating characteristic (ROC) curves comparing the performance of ABA and FCM in detecting fetal blood laced with blood from male adults. Blood samples were prepared by serially diluting umbilical cord (n=14) blood in blood of male adults (n=57) at dilutions 1/10, 1/100, 1/1,000, and 1/10,000, resulting in 285 artificially diluted blood samples, containing from 1 to 1,000 fetal red blood cells per 10,000 adult red blood cells.

The accuracy of FCM and ABA to detect fetal blood was evaluated by plotting the receiver operating characteristic (ROC) curves for each method (Figure 2 and Supplementary Figure 1). Overall, FCM (area under curve, AUC = 0.8723) had better performance than ABA (AUC = 0.6569) to detect the presence of fetal blood laced with blood of male adults (Figure 2). Additionally, ABA had lower specificity (59.65%) and lower negative predictive value (31.78%) compared with FCM (78.95% and 58.44% respectively), indicating that assessment of FMH by ABA could be implied in a high probability of false positive results (Supplementary Table 2).

### 3.2. Correlation and Agreement between FCM and ABA

**Figure 3:**
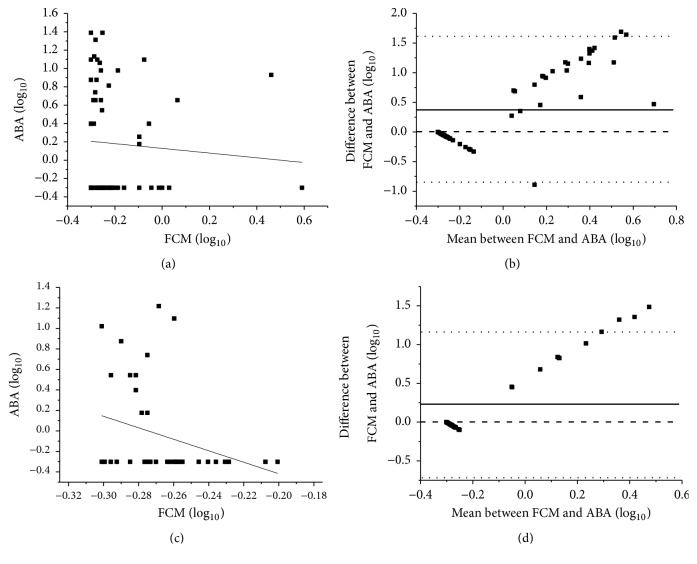
Parallel analysis of results obtained by FCM and ABA. (a) Low correlation (Spearman correlation: r = -0.263, p= 0.039, and n = 57) and (b) lack of agreement (bias d = 0.342, standard deviation = 0.6047, and confidence interval = 95% [0.842; 1.526] p = 0.559) were found for the results assessed in blood samples of male adults added with umbilical cord blood at dilutions 1/10, 1/100, 1/1,000, and 1/10,000. (c) No correlation (Spearman correlation: r = -0.316, p = 0.047, n = 40) and (d) agreement (bias d= 0.233, standard deviation = 0.481, and confidence interval = 95% [-0.727; 0.397] p = 0.573) were found for the results assessed in blood samples from puerperal women.

The correlation and agreement between results obtained through FCM and ABA were further compared following assessment of both blood samples of male adults containing fetal blood at different dilutions (processed as aforementioned) and blood from puerperal women taken within 48 hours following delivery. Thus, low correlation (Figure 3(a)) and lack of agreement (Figure 3(b)) were found between results obtained by FCM and ABA for samples of male adults containing fetal blood. Similarly, there was no correlation (Figure 3(c)) or agreement (Figure 3(d)) between results obtained by FCM and ABA for the contents of fetal blood in samples from puerperal women.

FCM and ABA were compared for screening of FMH in puerperal women, taking into consideration thresholds (Supplementary Table 2) previously specified for these methods while assessing blood samples from male adults containing fetal blood. As indicated in Table 2, most of the puerperal women were categorized below the threshold specified for the lowest dilution of fetal blood. At 1/10 dilution; for example, 80% of the puerperal women (32 in 40) were below the thresholds established for FCM and ABA. However, as dilution increased, numbers of puerperal women ranged among the different classes and revealed a disparity between the two methods; i.e. ABA and FCM disagreed in indicating the same set of puerperal women as being above the thresholds. Thus, at 1/10,000 dilution, ABA indicated 27.5% of the puerperal women (11 in 40) were above the thresholds while FCM indicated 17.5% (7 in 40), which comprised a different group of puerperal women (Table 2). Based on these results, it was determined that, at a critical level of 0.5%, ABA indicated that 27.5% of puerperal women would have FMH while FCM did not detect FMH.

## 4. Discussion

In this study, ABA was evaluated as a screening method for the detection of FMH. To our knowledge, this was the first time that such a method is systematically evaluated for this end. Additionally, accuracy of ABA to detect fetal blood was also evaluated and compared with FCM. Prevalence of isoimmunization due to RhD incompatibility between mother and her fetus has been lowered by the use of recommended prophylaxis [15]. However, FMH detection relies on methods with limited availability and, therefore, new automatized methods could benefit FMH diagnosis and treatment. Blood evaluation through ABA requires small amounts of blood and is less dependent on operator's practice and less expensive than FCM. Additionally, ABA is more accessible, since most of the intensive care units have this equipment [16].

Nevertheless, our results showed that evaluation of blood through ABA could be implied in overestimation of FMH. This feature is possibly a consequence of ABA's failure to differentiate between red blood cells from fetal blood and F-cells from adult blood. F-cells are red blood cells from adults containing HbF. Although these cells can frequently be found in low amounts in the blood of healthy adults, their numbers are increased in certain inherited hemoglobin disorders or in acquired conditions, such as acute erythropoietic stress and cancer [17–21]. It was also reported that F-cells can be increased during pregnancy as a result of physiological response [22]. However, Pastoret et al. [23] established 2.2% (0.3 to 11.3%) and 3.4% (0.9 to 13.6%) as reference values of F-cells in pregnant and nonpregnant women, respectively. Similarly, we have found 2.6% (0.0 to 9.9%) as reference value of F-cells in blood of male adult donors (data not shown).

Differently, detection of fetal red blood cells by FCM is based on the specific labeling of HbF by fluorophore-conjugated monoclonal antibodies (anti-HbF) [24, 25]. Additionally, anti-HbF specificity varies between HbF contained in fetal red blood cells and F-cells, favoring differentiation not only fetal red blood cells but also F-cells in blood of adults, as a consequence of differences in the resulting fluorescence intensity (Figure 1). Nevertheless, our data showed that anti-HbF caused variable amounts of positive events that were detected within the gate of fetal red blood cells in blood samples of male adults that were not added with umbilical cord blood. These positive events seem to be adult F-cells, at which the fluorescence intensity dragged them beyond the specific gate of adult F-cells. Although detected in low amounts, these positive events were responsible for certain loss of linearity of FCM, as dilution of fetal cells in blood samples of male adult increased (Supplementary Table 2).

FCM has been systematically evaluated for FMH diagnosis during the last decade and results have shown its advantages for assessment of fetal red blood cells and/or F-cells in blood samples [20, 22, 23, 26]. Improved FCM protocols employing anti-HbF in combination with other monoclonal antibodies, such as anticarbonic anhydrase or anti-RhD, were also evaluated [7, 27]. However, volume of fetal blood leakage in FMH is rarely calculated in daily practice and, therefore, standard doses of anti-D immunoglobulin, ranging from 250 to 300*μ*g, are administered [26, 28, 29]. These doses are based on the recommendation to use 25*μ*g of anti-D immunoglobulin for each ml of fetal blood leaked into the maternal blood compartment [30]. Without the accurate evaluation of FMH, this dose can represent an overtreatment. However, with the use of this protocol, only 0.3 to 1.0% of treatment fails when the standard doses of immunoglobulin are employed on the 28^th^ week of gestation and within 72 hours postpartum [21, 31, 32].

Finally, although the predictive positive value specified for ABA (87.08%, Supplementary Table 2) has initially suggested the ability of this method to detect fetal blood laced with blood from male adults, our results showed that ABA overestimates FMH and disagree with FCM on indicating puerperal women with FMH.

## 5. Conclusion

ABA is inadequate for being used to screen for or to measure FMH.

## Figures and Tables

**Table 1:** Characteristics of the subjects at time of blood sampling.

		Newborns	Adult males	Puerperal women
Sex	Female	8	-	40
	Male	6	57	-
Gestational age (weeks)		36 (33-41)	-	-
Age (years)		-	18-60	18-42
ABO group	A	5	11	15
	B	3	3	4
	AB	-	-	1
	O	6	46	20
RhD	Positive	14	56	36
	Negative	0	4	4

**Table 2:** Comparison between flow cytometry (FCM) and automatic blood analyzer (ABA) for screening of fetal maternal hemorrhage (FMH) in puerperal women (n=40). Thresholds were specified for FCM and ABA following the assessment of blood samples of male adults added with fetal blood. Blood samples were prepared by serially diluting umbilical cord blood in blood of male adults at dilutions 1/10, 1/100, 1/1,000, and 1/10,000.

	All dilutions				1:10				1:100				1:1.000				1:10.000			
		ABA				ABA				ABA				ABA				ABA		
	Threshold	< 0.500	≥ 0.500	Total	Threshold	< 2.500	≥ 2.500	Total	Threshold	<0.500	≥ 0.500	Total	Threshold	< 2.500	≥ 2.500	Total	Threshold	< 0.500	≥ 0.500	Total

FCM	< 0,0945	27	11	38	< 3,020	32	8	40	< 0,450	29	11	40	< 0,096	30	8	38	< 0,061	22	11	33
	≥ 0,0945	2	0	2	≥ 3,020	0	0	0	≥ 0,450	0	0	0	≥ 0,096	2	0	2	≥ 0,061	7	0	7
	Total	29	11	40	Total	32	8	40	Total	29	11	40	Total	32	8	40	Total	29	11	40

## Data Availability

The data used to support the findings of this study are available from the corresponding author upon request.

## Supplementary Materials

Supplementary Materials Supplementary Table 1. Comparison between blood samples of male adults with and without fetal blood. All samples were assed for the amounts of fetal red blood cells or fetal hemoglobin by flow cytometry (FCM) or automatic blood analyzer (ABA), respectively. Blood samples were prepared by serially diluting umbilical cord blood in blood from male adult donors at dilutions 1/10, 1/100, 1/1,000, and 1/10,000. Supplementary Table 2. Comparison of flow cytometry (FCM) and automatic blood analyzer (ABA) at thresholds that maximizes sensitivity and specificity by combining all blood samples independently of dilution, and for each of the fetal blood dilutions. Blood samples were prepared by serially diluting umbilical cord blood in whole blood of male adult donors at dilutions 1/10, 1/100, 1/1,000, and 1/10,000. Supplementary Figure 1. Receiver operating characteristic (ROC) curves comparing the performance of ABA and FCM in detecting fetal blood laced with blood from male adults at dilutions 1/10 (A), 1/100 (B), 1/1,000 (C), and 1/10,000 (D). Samples were prepared by serially diluting umbilical cord blood in whole blood of male adult donors. AUC = area under curve.
