# Supplementary material for: Evaluation of Automatic Blood Analyzer as Screening Method in Fetomaternal Hemorrhage
Source: Biomed Res Int. 2019 Feb 26;2019:6481654. doi: 10.1155/2019/6481654 (PMC6413365; doi:10.1155/2019/6481654)
Supplement: Supplementary Materials — Supplementary Table 1. Comparison between blood samples of male adults with and without fetal blood. All samples were assed for the amounts of fetal red blood cells or fetal hemoglobin by flow cytometry (FCM) or automatic blood analyzer (ABA), respectively. Blood samples were prepared by serially diluting umbilical cord blood in blood from male adult donors at dilutions 1/10, 1/100, 1/1,000, and 1/10,000. Supplementary Table 2. Comparison of flow cytometry (FCM) and automatic blood analyzer (ABA) at thresholds that maximizes sensitivity and specificity by combining all blood samples independently of dilution, and for each of the fetal blood dilutions. Blood samples were prepared by serially diluting umbilical cord blood in whole blood of male adult donors at dilutions 1/10, 1/100, 1/1,000, and 1/10,000. Supplementary Figure 1. Receiver operating characteristic (ROC) curves comparing the performance of ABA and FCM in detecting fetal blood laced with blood from male adults at dilutions 1/10 (A), 1/100 (B), 1/1,000 (C), and 1/10,000 (D). Samples were prepared by serially diluting umbilical cord blood in whole blood of male adult donors. AUC = area under curve. [file 6481654.f1.pdf]

Supplementary Materials

Supplementary table 1. Comparison between blood samples of male adults with and without fetal blood. All samples were assed for the amounts of fetal red blood cells or fetal hemoglobin by flow cytometry (FCM) or automatic blood analyzer (ABA) respectively. Blood samples were prepared by serially diluting umbilical cord blood in blood from male adult donors at dilutions 1/10, 1/100, 1/1,000 and 1/10,000.

	**Dilution of cord blood in adult blood**				
	**0 (n=57)**	**1/10 (n=57)**	**1/100 (n=57)**	**1/1,000 (n=57)**	**1/10,000 (n=57)**
**FCM**	0.04 (0.00-2.93)	7.09 (1.98-15.30) *p<0.0001*	1.13 (0.00-2.52) *p<0.0001*	0.16 (0.00-0.95)	0.04 (0.00-0.96)
**ABA**	0.00 (0.00-24.00)	7.00 (0.00-21.00) *p<0.0001*	1.00 (0.00-11.00)	0.00 (0.00-9.00)	0.00 (0.00-8.00)

Values are shown as median and range. Results of the serially diluted blood samples were calculated after correction of the data by subtracting the values obtained in blood samples of male adults. $p<0.05$ indicates significant statistical difference compared with column "dilution 0", assessed by t-Student test. (FCM = % Fetal red blood cells in total cells and ABA = % Fetal hemoglobin in total hemoglobin).

Supplementary table 2. Comparison of flow cytometry (FCM) and automatic blood analyzer (ABA) at thresholds that maximizes sensitivity and specificity by combining all blood samples independently of dilution, and for each of the fetal blood dilutions. Blood samples were prepared by serially diluting umbilical cord blood in whole blood of male adult donors at dilutions 1/10, 1/100, 1/1,000 and 1/10,000.

**ROC curve**	**All Samples**		**1:10**		**1:100**		**1:1.000**		**1:10.000**	
	FCM	ABA	FCM	ABA	FCM	ABA	FCM	ABA	FCM	ABA
Threshold	≥ 0.0945	≥ 0.5000	≥ 3.020	≥ 2.50	≥ 0.4500	≥ 0.5000	≥ 0.0965	≥ 2.500	≥ 0.0610	≥ 0.5000
Sensitivity (%)	85.96	67.98	100.0	87.72	91.23	64.91	87.72	57.89	68.42	56.14
Specificity (%)	78.95	59.65	100.00	68.42	91.23	59.65	78.95	59.65	70.18	59.65
Positive predictive value (%)	94.23	87.08	100.00	73.53	91.23	61.67	80.65	58.93	69.64	58.18
Negative predictive value (%)	58.44	31.78	100.00	84.78	91.23	62.96	86.54	58.62	68.97	57.63

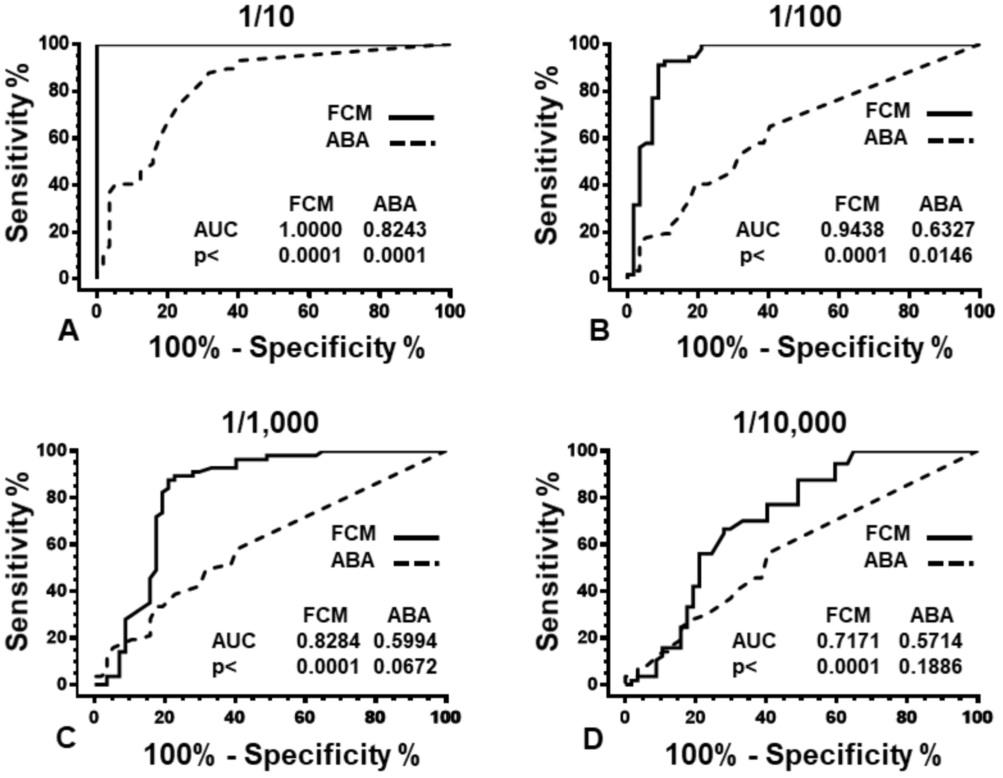

Supplementary figure 1. Receiver operating characteristic (ROC) curves comparing the performance of ABA and FCM in detecting fetal blood laced with blood from male adults at dilutions 1/10 (A), 1/100 (B), 1/1,000 (C) and 1/10,000 (D). Samples were prepared by serially diluting umbilical cord blood in whole blood of male adult donors. AUC = area under curve.
